# Proposal for a new conceptual framework to guide early intervention professionals in a transcultural context

**DOI:** 10.3389/fpubh.2025.1689596

**Published:** 2025-12-12

**Authors:** Berna Elias, Aline Bogossian

**Affiliations:** 1 School of Social Work, Faculty of Arts and Science, University of Montreal, Montreal, QC, Canada; 2 Lethbridge-Layton-Mackay Rehabilitation Centre, Centre for Interdisciplinary Research in Rehabilitation of Metropolitan Montréal, CIUSSS West-Central Montreal, Montreal, QC, Canada

**Keywords:** early intervention, partnership, parental engagement, transcultural context, neurodevelopmental conditions, anti-oppressive approach, immigrant parents

## Abstract

**Background:**

Parental engagement in early intervention programs for children with neurodevelopmental conditions is complex, particularly within transcultural contexts. This study examines current models of parental engagement to identify key factors that support inclusive approaches and to develop a flexible framework that addresses the realities of immigrant families raising children with neurodevelopmental conditions in transcultural settings.

**Methods:**

A critical nonsystematic review was conducted to integrate parental engagement models across multiple fields, aiming to develop a theoretical tool to guide a doctoral research project.

**Results:**

We found that current models often ignore transcultural realities and power dynamics. Therefore, we propose the Child–Parent-Professional Partnership framework, which provides a holistic ecological approach sensitive to the realities of immigrant parents.

**Limitations:**

The findings presented are inherently tentative and serve as a foundational basis for the empirical research design subsequently undertaken by the first author. Accordingly, the present article is intended to serve as an initial conceptual exploration, rather than a definitive analysis, and further empirical investigation is necessary to validate these primary insights and extend their scope.

**Conclusion:**

The proposed framework provides a comprehensive view of the influences affecting parental engagement in transcultural contexts, emphasizing the importance of an anti-oppressive approach to foster partnership that are attuned to the realities of immigrant parents. The originality of this framework lies in its integration of theoretical and practical approaches, offering both a conceptual lens to understand complex dynamics and a foundation for implementing authentic partnerships.

## 1 Introduction

Parental engagement (PE) in early intervention (EI) programs for children with neurodevelopmental conditions (NDC) is crucial for optimal developmental outcomes. However, the concept of parental involvement or engagement can be loaded and can lead to misunderstandings and confusion (1) about what it might look like in practice, and what professionals can do to support the engagement of parents in EI. A diversity of terms related to PE exists in the literature (50) and they often do not accurately represent the inherent dynamics that exist in interpersonal relationships (such as power dynamics between parents and professionals) particularly within a transcultural context. In other words, existing models often overlook transcultural realities and power dynamics. To address these gaps, we conducted a literature review aimed at developing a new conceptual framework that highlights key factors for building equitable partnerships with immigrant families of children living with NDC in EI programs. This comprehensive framework integrates critical theoretical and bioecological perspectives, shifting the focus found within existing models of PE to a broader framework of child–parent-professional partnership (CPPP) within a transcultural context.

This CPPP framework was developed following a critical literature review (2) conducted by the first author as a foundational phase of her doctoral thesis project. A purposive unsystematic review approach was selected to allow for broader reflection on PE as it has been represented in diverse fields to collect insights that would otherwise be missed through other approaches (3). The purpose of this review was to examine broadly how the concept of PE has been defined in existing models, to review and document the components that make up those models, and to draw on the most relevant to develop a new framework that better reflects and address the unique realities of immigrant families of children living with NDC in transcultural contexts. In other words, the emphasis of this work was to conduct profound inquiries and generate original insights, rather than define PE comprehensively, or assess the overall evidence base (3).

This framework, presented as tentative and hypothetical, offers a promising new lens for bridging theory and practice for a population that is rarely considered. The initial conceptual exploration represented in this article was a primary phase toward developing a *practice theory*^1^ that was subsequently applied in fieldwork conducted as part of the first author’s doctoral research. As Payne (4) notes, frameworks are one approach within practice theory; they serve as guides by organizing knowledge and outlining a range of approaches for addressing practice situations. Therefore, the originality of this framework lies in its integration of theoretical and practical approaches, offering both a conceptual lens to understand complex dynamics and a foundation for implementing authentic partnerships.

Furthermore, rooted in social work, it is intended to support interdisciplinary teams by expanding beyond models that are conceptualized in disciplinary silos. Unlike traditional approaches that focus primarily on micro-system factors, this framework incorporates broader environmental influences, allowing for nuanced analysis of various factors affecting the development of collaboration and partnership. Embedded in the bioecological model (5), and informed by both transcultural perspectives (6–8) and anti-oppressive approaches (9, 10). This integrative approach will enable researchers and professionals to consider the complex interplay of individual, familial, cultural, and systemic factors influencing parent-professionals partnership in EI programs.

## 2 Parental engagement in early intervention

We draw on the following definition of parental engagement as “the active involvement and investment of the parent or other primary family caregiver in the intervention process” (11), p. 1. In the context of EI programs^2^ for children with NDC, parental engagement is an active ingredient for achieving optimal developmental outcomes. Parents are considered key actors in their children’s lives and in fostering children’s development (12). They play complex roles in EI programs; they are expected to learn about their child’s diagnosis, acquire new skills, and are often guided or coached to apply intervention strategies to support and encourage the consolidation of new skills at home.

Research has demonstrated the benefits of PE across various domains, including educational program outcomes (13–15), family support programs (16, 17), mental health (18–20) and EI programs (21, 22). While PE is crucial, it can be challenging to achieve and maintain (18, 19). Factors influencing PE include parents’ perceptions of therapy’s value and success, positive expectations and interpersonal relationships (23). Additionally, professionals’ understanding of families’ social conditions affects engagement and intervention outcomes (24).

## 3 Parental engagement in a transcultural context

Canada’s ethnocultural diversity presents unique challenges and opportunities for PE in EI programs. To better understand PE in this context, it is essential to consider the transcultural context, characterized by the complexities of cultural transitions, the migratory journey, the immigration process and their impact on families’ lives (6). These transitions often involve the deconstruction and reconstruction of identity to adapt to a new life far from usual support networks. The immigration process can destabilize parents and require psychological and cultural reorganization as they integrate a new multi-ethnic, linguistic, and cultural environment. Immigrant parents of children with NDC will face additional complexities including differing values and visions of parenting, child development and rehabilitation needs. This cultural diversity, shaped by various belief systems and immigration patterns, can create significant disparities in understanding between parents and professionals working with families. These disparities may result in pronounced contradictions, leading to misalignments in expectations, goals and intervention strategies, potentially impacting the effectiveness of intervention. The context of immigration may also foster opportunities for dynamic cultural exchange between parents and professionals, where both parties contribute to and learn from each other’s perspectives (25). It is crucial to recognize that the migratory journey itself along with beliefs, customs and practices that immigrants bring with them, significantly shapes the context of intervention (6). Professionals must acknowledge and integrate these elements into their approach to ensure culturally responsive support and refrain from the simplistic categorization of people into cultural classifications.

## 4 Theoretical background

Our investigation is grounded in the bioecological model (5) as the predominant theoretical influence, complemented by two intervention approaches: transcultural perspectives (6–8), and anti-oppressive approaches (9, 10). The bioecological model serves as the foundational theoretical framework allowing for the examination of human development from an interdisciplinary and integrative perspective. It enables consideration of individuals in their complexity and in interaction with proximal and distal social systems over time and space, facilitating the integration of objective and subjective elements across various systemic layers.

Transcultural perspectives (6–8) enhance our understanding of diverse identities interacting within EI programs by recognizing the impact of cultural backgrounds of both professionals and parents on intervention processes. The impact of migratory journeys on family wellbeing is also a salient consideration, as are the beliefs, traditions and changes in cultural references that shape immigrant family’s life (6). Consequently, professionals aiming to create impactful interventions must prioritize and center the family interpretation of their situation, which may inherently be shaped by their unique migration and settlement experiences. This approach acknowledges that families are the experts of their own experiences and that their cultural perspectives are integral to the intervention process.

Anti-oppressive approaches (9, 10) form the third pillar of our theoretical framework. These approaches emphasize the importance of addressing power imbalances and systemic inequities that may affect intervention. They recognize and challenge structural barriers that immigrant families may face in benefiting from services, promote equity and social justice within the intervention process (26), and encourage professionals to critically examine their social positioning, professional posture, and power dynamics in interpersonal relationships (27).

These three theoretical approaches underpin our work and provide a comprehensive lens through which to examine the complex interplay of factors that influence PE for immigrant families. They inform our analytical perspective and have shaped the design of the proposed framework. These choices reflect the need to capture diverse perspectives of immigrant families and professionals, to examine multiple systemic layers influencing intervention outcomes, and to address power dynamics within both the research and intervention process.

## 5 Materials and methods

This article presents work conducted by the first author in a primary phase of her doctoral research project in which a literature review was a requirement to develop a future doctoral empirical study. The purpose of this primary phase was to explore how the concept of PE has been addressed in the literature, with an aim to examine the key elements within models of parental engagement and critically examine how these reflect and address the unique realities of immigrant families of children with NDC in transcultural contexts.

We employed a critical review methodology (2) in a non-systematic review approach (3, 28). The latter, or “[p]urposive reviews tend to address broader, far-reaching, and less defined questions. They seek to integrate findings across fields, often focusing on ‘Why or how it works?’ in addition to the simpler ‘Does it work?’” (3), p. 58.

**Figure 1:**
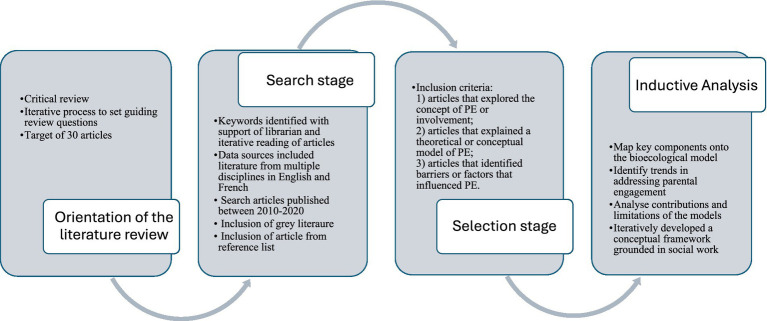
The review process.

Therefore, the present review is grounded in a constructivist paradigm drawing on qualitative research methodologies and intentionally designed to be flexible, creative, iterative, and reflective (29). This flexibility distinguishes critical reviews from other more structured forms of literature reviews. The adaptable nature of this approach does not adhere to a systematic strategy and instead invites researchers to draw on their expertise and knowledge when selecting and evaluating literature. It allows researchers to develop nuanced and contextualized interpretations of the materials that may, in turn, potentially culminate in the creation of novel conceptual frameworks (29). Figure 1 provides an overview of the review process.

### 5.1 Guiding questions

The following questions guided our review and analysis of published materials:

1. How has the concept of ‘parental engagement’ been addressed in the literature?
2. How can key elements identified in this review be integrated in a new framework to reflect and address the unique realities of immigrant families of children with NDC in transcultural contexts?

### 5.2 Search strategy

The process of our critical review goes beyond demonstrating systemic research and merely describing existing literature; it involves a thorough analysis that may result in conceptual innovation, a new model, or a theory (2). As Cook (3) asserts, unsystematic or purposive reviews have the capacity to accommodate the complexity inherent in social interventions and interactions. Therefore, our search strategy was “intentionally selective rather than systematic,” allowing for a more focused and purposeful examination of relevant literature (29), p. 187. The search strategy was conducted using a creative approach, as recommended by Snyder (30). This approach deviates from the conventional practice of covering every existing publication on a subject and following a systematic screening process. As an alternative, it prioritizes the integration of diverse perspectives and insights from multiple disciplines or research traditions. To this end, and in order to capture the complexity of interpersonal and social dynamics that occur in EI, a selective literature search was conducted to identify articles in which conceptual models of parental involvement/engagement were presented from various fields of study including intervention (EI, mental health, rehabilitation), education, family support and child welfare.

#### 5.2.1 Data sources/materials

We employed a search strategy that aimed to encompass databases covering research in social work and allied health, education, and social sciences. These databases included Social Services Abstracts, Sociological Abstracts, EBSCO, Social Work Abstracts (primarily English publications), and CAIRN and ÉRUDIT (primarily French publications). Our search involved testing a combination of keywords in French and English (for example, engagement, involvement, partnership, collaboration with parent*, family, father*, mother*) based on recommendations from a university librarian. We examined articles published between 2010 and 2020 that appeared most relevant to the first author’s doctoral project addressing parental involvement and/or engagement. This time frame^3^ was considered appropriate for the purposes of the doctoral project and yielded a significant number of sources to be explored and selected. The search strategy also involved explorations of gray literature and reference lists of relevant articles.

#### 5.2.2 Target sample

As Kahlke et al. (29) state “…critical reviewers are inevitably limited to sharing only a sample of the sources they encountered.” (p.187). In fact, the process of exploration is complete once the objective of generating a meaningful synthesis has been attained, thereby providing a valuable contribution to the field. A target of 30 articles was set for this critical review. The primary author examined a broad range of studies and selected the 30 most relevant articles, representing a broad range of disciplines and models, for this review. The sampling method diverged from conventional systematic screening and focused on retaining studies illustrative of the broader field, as suggested by Paré and Kitsiou (31) and Cook (3). The articles originated from a broad range of fields and subspecialties, including articles from educational settings, various intervention fields (for example, mental health, rehabilitation, home visit), family-centered approaches & child welfare, and gray literature.

### 5.3 Selection stage

Decisions regarding the inclusion and exclusion of articles are nuanced, strategic, adaptable and characterized by their relevance to the guiding questions (3, 29). The selection strategy was informed by the author’s considerable experience working with individuals living with NDC, along with their families and practitioners supporting them in both the education and rehabilitation sectors. A non-systematic approach characterized by a strategic and selective incorporation of data was adopted (3). The articles that were considered for inclusion in the review were those that:

- explored the concept of PE or involvement;
- explained a theoretical or conceptual model of PE;
- identified barriers or factors that influenced PE.

The objective was to identify their contributions and limitations, with the hope that this may lead to a new comprehensive framework.

### 5.4 Analysis process

According to Cook (28), non-systematic approaches frequently employ inductive analysis and rely on perceptive synthesis. Researchers prioritize perceived relevance to the study issue and deliberately incorporate a variety of sources that are considered opportunities for novel perspectives. The analysis typically adopted is of a qualitative and narrative nature, yielding rich, critical, and interpretative outcomes.

Firstly, the concepts and elements comprising each PE model were identified and critically reviewed. These elements were then mapped onto the bioecological model, allowing for a systematic organization of factors involved in PE models across different ecological levels (micro, meso, exo, macro). Subsequently, an exhaustive investigation into trends in the conceptualization and treatment of the PE concept was conducted. This encompassed a meticulous examination of selected models and articles. Subsequently, an analysis was undertaken to ascertain the contributions and limitations of extant literature. Based on the insights from the literature, a set of guiding principles was developed to hypothesize a conceptual framework that incorporated core elements of established PE models, concepts from transcultural perspective and principles from anti-oppressive approaches.

The framework has undergone validation by the comprehensive exam committee. It was also presented at multiple scientific conferences and to multidisciplinary professionals in various settings, where it was noted to be resonant by parents and professionals, and generated interest for its clear and simplified way of explaining and illustrating the interpersonal and social dynamics at play in EI.

## 6 Results

In this section we present the results of our critical literature review, organized around three main themes that address our first research question which concerns the ways in which PE has been addressed in the literature. First, we present trends in the exploration of PE. Second, we analyze the characteristics of PE as presented in the reviewed studies. Finally, we offer a critical discussion of the results, highlighting limitations of current approaches. This will be followed by an answer to the second question in the section that is intended to explain the new hypothetical conceptual framework.

### 6.1 Trends in the exploration of parental engagement

Our analysis revealed three primary trends in how the concept of parental engagement is approached in the selected literature (Supplementary material Table 2).

#### 6.1.1 Disciplinary silos in PE research

PE has been studied across various disciplines, this has led to broad variability in terminology, definitions and models (24, 32, 33, 50). The discourse on PE is extensive but often hindered by inconsistent terminology across various models and fields of study. The lack of a common definition and the multiplicity of models may cause confusion. This siloed approach can lead to misconceptions, as PE cannot be considered in isolation across different aspects of a child’s life. For example, we cannot consider PE in schools and education and ignore it when it occurs in rehabilitation programs for a child with NDC; it is crucial to look at the full picture.

#### 6.1.2 Narrow focus on factors that affect parental engagement

The second trend evident in the literature is a narrow focus of engagement within specific levels of the bioecological system. While PE has been examined at various contextual levels, most of the studies reviewed concentrate on only one or two of those levels. PE is examined at the micro and meso levels, with a focus on interactions between individuals, for instance in parent-professional encounters. The macro level is rarely considered in PE models, and few models integrate constructs across all levels (micro, meso, and exo). This limitation fails to provide a holistic view of the various factors influencing PE, as described in the bioecological model (5). To address these gaps, our analysis aims to consider both proximal and distal factors affecting PE, to map these factors onto the bioecological model, and include often overlooked macro-level influences such as immigration status and policies that can indirectly affect PE and work with immigrant families.

#### 6.1.3 Variability in delineating parental engagement

Researchers approach PE from different angles, focusing on levels, factors, components, processes, dimensions or conditions affecting engagement. A series of models have been designed to rationalize the concept and its components (See Supplementary Table 1 for a list of models identified in the selected literature). PE has also been explored in various contexts, highlighting both barriers and benefits within the distinct programs and specific settings (14, 19, 22). This diversity in delineating the concept makes it challenging to compare the literature and the models. Based on the current scholarly literature, there is no ‘one size fits all’ model of PE to guide practice and research with families. The most resounding need is for adaptable models that can meet the changing needs of families, responding to changes in a child’s development and conditions and accommodate the diverse settings the child may encounter (e.g., school, intervention program, para-scholar activities).

### 6.2 Characteristics of parental engagement

We have been able to identify some key elements that contribute to the delineation of the concept. Our analysis of the literature reveals four fundamental characteristics of PE (Supplementary material Table 3).

#### 6.2.1 Process-oriented and multifaceted nature

PE is widely recognized as a dynamic process involving multiple interacting factors (1, 19, 24, 32–34) and characterized by a multifaceted set of conditions (12, 19, 32, 35–37). It extends beyond simple indicators like attendance and adherence, encompassing factors that interact and are interdependent and thereby rendering engagement more complex. The examined models demonstrate a link to personal factors (e.g., education, affective stability, health conditions), to program-related factors (e.g., the intensity of the intervention, the environmental context, accessibility) and societal factors (e.g., social norms and values, policies).

#### 6.2.2 Contextual and complex interactive dynamics

PE operates within a complex ecosystem involving multiple actors, primarily practitioners and parents. This dynamic is influenced by family characteristics, interparental dynamics and organizational context such as program accessibility and implementation (24, 38).

The process of PE comprises six interrelated components: parent’s feelings; skills; knowledge; logistics; values and beliefs; and the parent-professional relationship (24). To achieve effective PE therapists are required to be well-trained, confident, and self-efficient (12, 39). This illustrates the interconnectivity between the various components of PE, as well as the complex dynamics of interaction that occur within a context involving multiple actors (child, parents, and professionals).

#### 6.2.3 Continuum of engagement

PE should be seen as a continuum. Goodall and Montgomery’s model (2014) illustrates the progression from initial involvement to the optimal level of engagement. The actions and responsibilities within PE change progressively and are renewable, adaptable to the development of the child and their evolving needs (40). Carman et al. model (2013) demonstrates that engagement can occur at different levels: direct care, organizational design and governance, and policymaking. In this model, engagement evolves along a continuum, beginning the time of initial consultation (the continuum lower end), progressing through the involvement point (the midpoint), and ultimately reaching partnership and shared leadership (the end of the continuum).

#### 6.2.4 Partnership-based approach

PE is increasingly framed as a partnership between families and professionals. Rivard et al. (22) define partnership “as the way in which families and service providers work together by building on each other’s expertise and resources to make joint decisions that will directly benefit the child and indirectly benefit the family and professionals.” (p. 4). Similarly, Roose et al. (41) framed PE as a bidirectional and reciprocal engagement between parents and professionals. They advocate for a democratic approach, highlighting the importance of reciprocal engagement and joint decision-making between parents, professionals and children.

### 6.3 Critical discussion

Our analysis of the most frequently cited characteristics in the literature has revealed several areas that require further attention to enhance understandings of factors influencing PE in interventions, particularly within a transcultural context. We have identified five key themes that warrant deeper consideration (Supplementary material Table 4).

#### 6.3.1 Limited focus of the role of other key players

Current models often overlook the roles of important factors beyond parents and professionals, such as those associated with the child (i.e., complexity of the diagnosis, age, needs, etc.) or those associated with the extended family members (i.e:, siblings, grandparents, fathers) or other key-actors (i.e., community leaders or cultural mediators). As Platt indicated that “the primary client of services is the child, and yet the focus of much concern about cooperation and engagement is the parent” (32), p. 140.

#### 6.3.2 Insufficient representation of reciprocal interdependence

The complex dynamic of interactions between various stakeholders are not adequately captured in existing models, particularly the mutual influence between parents, children and professionals. Some exceptions exist including those that consider the interparental dynamic (38), the parent–child dynamics (40) and inter-institutional partnerships (22).

#### 6.3.3 Narrow contextual framework

Many models fail to consider broader societal and cultural factors that can significantly influence the engagement process, such as immigration policies, socioeconomic factors and cultural norms. These factors can affect decision-making and stress levels (42, 43). While cultural influences in the engagement process were not specifically studied in the models we reviewed, some aspects such as external determinants or internal factors (e.g., the cultural competencies or the beliefs and perceptions) have been studied.

#### 6.3.4 Inadequate representation of family experiences

Current models oversimplify the diverse and complex experiences of families, especially those from different cultural backgrounds or with unique challenges. These unique experiences are more difficult to capture but must be considered when assessing the degree of engagement of parents.

Parents of children with NDC face significant emotional and physical challenges, including higher stress levels and health issues (44). These challenges are compounded by complexity of the child’s diagnosis and complexity of the family socioeconomic conditions (24). Understanding these processes can create conditions for more reasonable expectations of involvement, partnership and collaboration.

The unique experiences of immigrant families are not addressed in existing models. The adaptation process that immigrant families go through before experiencing a sense of belonging and understanding of the different systems that embrace the host society (i.e., socio-political, health and social services, legal, etc.) is also not taken into consideration.

#### 6.3.5 Terminology and responsibility issues

There is a need to critically examine the language used in PE models and reconsider how responsibilities are allocated among various stakeholders in the engagement process. Based on an anti-oppressive approach, we reflected on how the term ‘parental engagement’ places the burden of collaboration on only one actor in this complex dynamic of interaction: the parent. However, the literature discussed above refer to collaboration between different actors (i.e., parents, professionals, and child). It is therefore pertinent to question the continued use of the term ‘parental engagement’ in this context, and its subtle impact on the existing power dynamics within the intervention.

## 7 From parental engagement to child–parent-professional partnership

Drawing insights from our critical literature review, we observe that the concept of parental engagement in EI programs has gained significant attention across various disciplines. However, existing models do not always capture the full complexity of this dynamic process. We propose the Child–Parent-Professional Partnership (CPPP) framework, a concept derived from a comprehensive review of extant literature and critical analysis. This framework addresses the limitations of previous approaches by offering a nuanced understanding of engagement in EI within transcultural contexts.

The CPPP framework conceptualizes engagement as a dynamic and complex partnership process involving three primary actors (parents, children and EI professionals). Rooted in Bronfenbrenner & Morris’s bioecological model of child development (2006), the framework recognizes that engagement is shaped by reciprocal influences among various stakeholders (i.e., children, parents, and professionals), and across the different levels (micro, mezzo, exo, macro).

A key feature of the CPPP framework is its adaptability, evolving in response to changing developmental needs. The framework posits that engagement exists on a continuum ranging from consultation to full partnership, with the latter representing the ideal goal, fostering ownership and commitment among all stakeholders. Guided by democratic governance principles and equitable relationships among all participants, the CPPP framework allows for flexible distribution of roles and responsibilities; accommodating the specific conditions, objectives, and mutual agreements of all involved in the dynamic partnership.

### 7.1 An adapted framework for a holistic view

The CPPP framework builds on existing EI program insights, emphasizing a patient and family-centered approach at the micro and meso levels, where family-child-practitioner collaboration occurs. This proposed theoretical framework is characterized by its consideration of a broader contextual setting, allowing for the analysis of various components, factors, dimensions and conditions that might influence this partnership over time. The analysis undertaken within a multi-level system comprising bio-psycho-socio-cultural domains, is intended to facilitate identifying elements that may hinder or enhance the evolving engagement process.

#### 7.1.1 Central components of the CPPP framework

**Figure 2:**
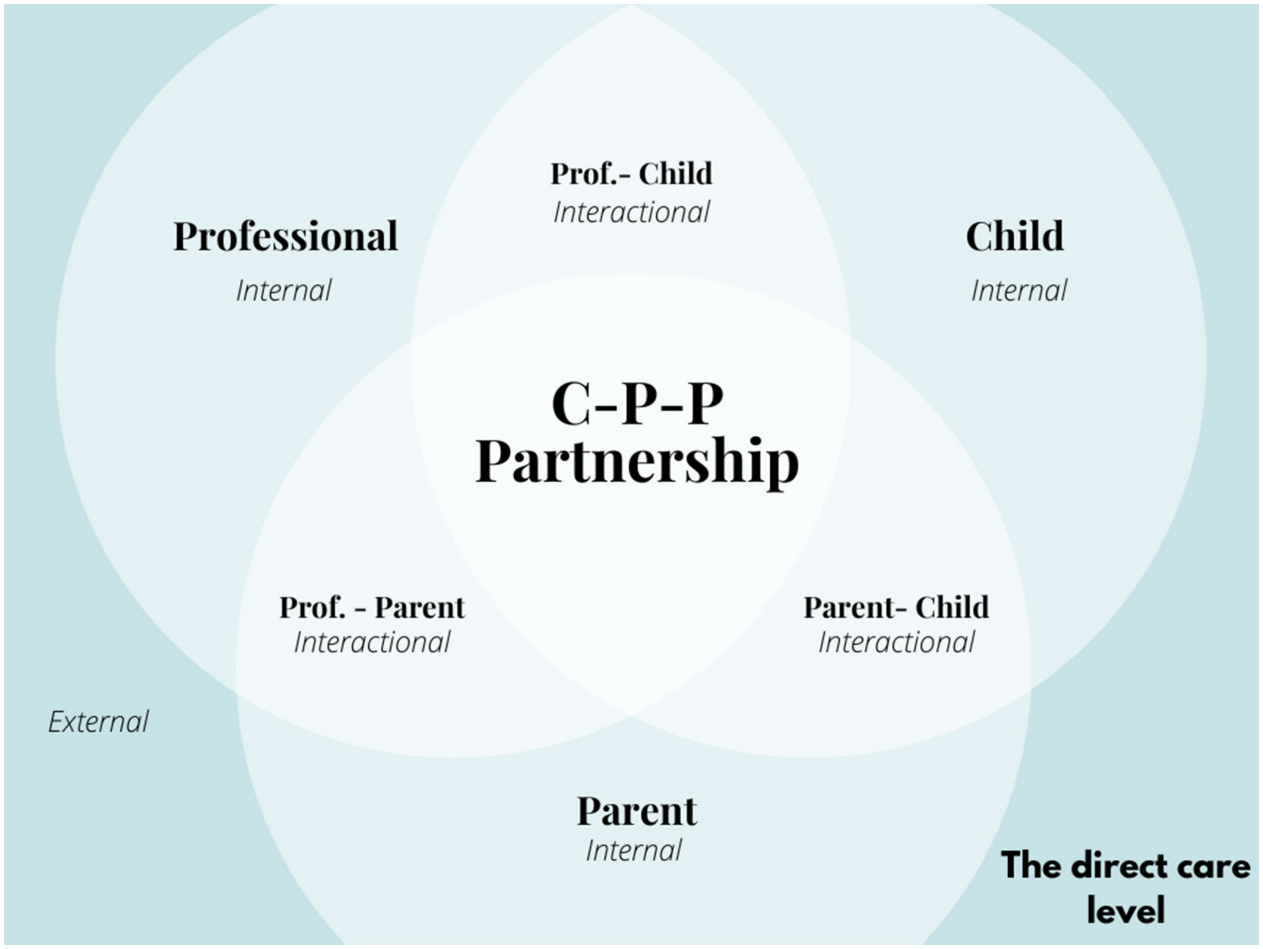
Direct care level dynamic between Child-Parent-Professional.

Figure 2 illustrates the initial phase of our analysis of the complex dynamic of interactions, representing the central part of the CPPP, the direct care level where the partnership between the child, the parent and the professional takes place. Each actor is influenced by a series of internal, external, and interactional dimensions.

This simplified illustration, which focuses on the three primary actors, serves as a useful reminder to professionals about their role in this dynamic process. It is crucial to recognize that professionals’ internal, external and interactional factors are as present in this dynamic as those related to the parent or child. Professionals may also need to consider the involvement of additional actors (siblings, other professionals, etc.) who could play a part in the partnership. An individualized perspective at the micro level is imperative to evaluate who should be included in this ecosystem (49).

Figure 2 consists of three circles, each representing a different partner in the relationship: the child, the parent, and the professional. The intersection of the circles illustrates the partnership. Each circle represents a different internal dimension characterized by specific attributes, such as cognitive, affective, and behavioral factors. The intersection of two circles symbolizes the interactional dimensions emerging from the relationships between two partners: child-professional, child–parent, and parent-professional. These interactional dimensions can encompass factors such as the working alliance, communication, trust, negotiation, and many others. Finally, external factors stem from the surrounding context (e.g., institutional, cultural, socioeconomic, political) and exert a profound influence on the partnership. This visual illustrate the partnership’s complexity and dynamics by highlighting individual contributions, mutual interactions, and contextual influences.

#### 7.1.2 Key dimensions of the CPPP framework

##### 7.1.2.1 Internal dimensions

These encompass factors related to personal dispositions of everyone involved in the partnership. For parents’, internal factors include affective, cognitive, behavioral and health conditions, combined with personal experiences that shape identity and motivation, as well as components such as receptiveness, willingness, self-efficacy, and expectations (12, 24, 32, 49). For professionals, internal factors are similar to those of the parents, but also include professional expertise, which influences decision-making and interaction quality. For children, internal factors relate to the complexity of the case or diagnosis (e.g., cognitive or/and developmental competencies and achievements) as well as the health, psychological, and behavioral dispositions.

##### 7.1.2.2 External dimensions

These refer to the contextual elements of each partner that go beyond individual control. For parents, these include family conditions (economic situation, cultural values and beliefs, the (un)availability of their support network), accessible resources and logistic constraints [the service complexity, policies and program structures (24, 32)]. For professionals, external factors include societal conditions, institutional culture, systemic logistics, and organizational structure. For children, external factors involve all the above.

##### 7.1.2.3 Interactional dimensions

These factors capture the relational dynamics between the partners. Described in the bioecological model (5) at the mesosystem level, these dimensions represent interactions between different entities that directly impact the child. Key interactional dimensions include: (a) Parent-professional(s) relationships; (b) Child–parent interactions; (c) Child-professional interactions; (d) Parent–parent (or the interparental dynamic) and other relationships directly affecting the child.

It is important to note that this is a simplified initial version of the model, designed to highlight the core interactional dimensions while acknowledging the potential for including additional stakeholders and relationships.

By considering these dimensions, our hypothetical conceptual framework provides a comprehensive perspective on factors that may impact the partnership between the main actors within a complex dynamic of interactions. While it may not encompass all areas, it offers a broader view of potential interferences in the process, tailored to the unique realities of each family.

### 7.2 Child–parent-professional partnership embedded in the bioecological model encompassing the transcultural context

**Figure 3:**
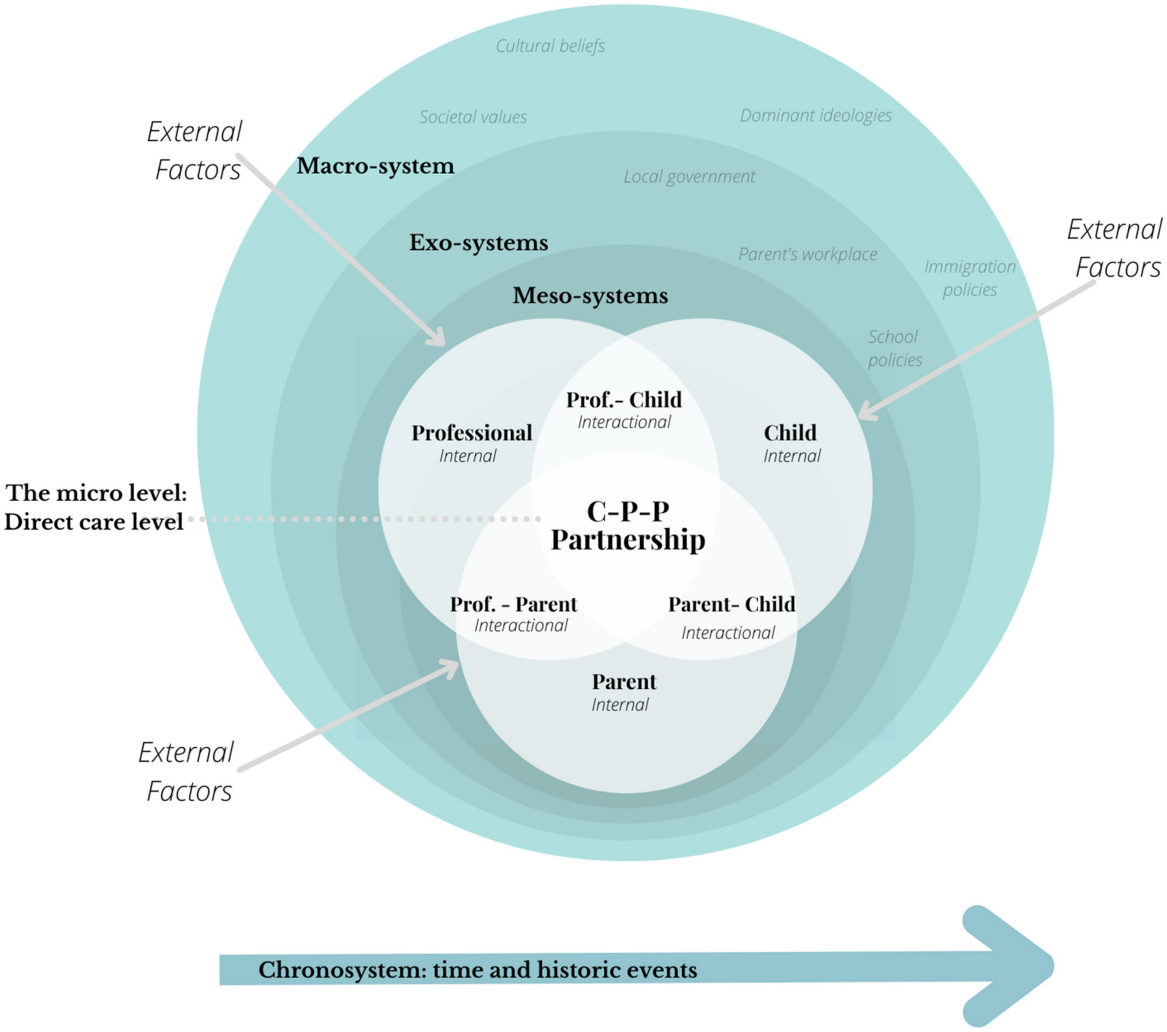
The CPPP embedded in the bioecological model.

The CPPP framework, embedded within the bioecological model, offers a holistic ecological perspective to understand immigrant families’ conditions in a transcultural context. It focuses on influencing factors at the micro level while maintaining broader observations at the macro level. To gain a more comprehensive understanding of the external dimensions and the conditions surrounding the child, parents, and professionals in a transcultural context, Figure 3 places the CPPP (Figure 2) at the heart of the bioecological model.

The CPPP framework resides at the heart of the bioecological model, occupying the meso level. This level represents the space where direct interactions between the various partners take place, illustrating the relational dynamics essential to the partnership.

Around this core, the exo level encompasses systems that, while indirectly related to the partnership, influence each of the partners. These include, for example, the parents’ workplace, school policies, the neighborhood, and provincial health service policies. These may affect a partner indirectly and, in turn, impact the quality and nature of the partnership. The macro level encloses the entire system. It represents broader societal factors, such as cultural values, dominant ideologies, federal policies, and economic trends. These influences, although more distant, shape the systems at the exo level and, as a result, influence the partners and their collaboration. Finally, the chronosystem introduces the dimension of time and historicity. It takes into account changes that occur over time, both at the individual level (child development, family transitions) and at the societal level (political developments, transformations in health or education systems). This dimension prompts us to examine the unique trajectories of all involved and how the dynamics of the partnership evolve over time.

This placement of the CPPP framework, at the heart of the bioecological model, allows for better analysis and addressing of multifaceted influences on the partnership and the child development, especially in transcultural context. This approach offers several advantages. It allows a more nuanced comprehension of the dynamic and complex nature of the influences, recognition of its adaptability to a child’s evolving needs over time, and consideration of a broader range of contextual factors, including cultural values, policies, and structural elements. Additionally, it fosters appreciation for the interactional dimensions within the partnership. However, it is important to note that the framework alone does not provide professionals and researchers with the necessary tools to establish effective partnerships. Consequently, adopting an anti-oppressive approach is crucial from a social work standpoint.

### 7.3 Requirements for an authentic CPPP: embracing an anti-oppressive approach

To embrace an anti-oppressive approach, it is essential to acknowledge multiple systems of oppression within a context and to adopt a reflective and reflexive practice^4^ (45). Building genuine partnerships means professionals need to move beyond the medical model of interaction where they act solely as experts defining problems and treatments, and instead recognize the importance of collaboration and shared decision-making with families. This aligns with the recommendations to redefine power differentials between professional workers and service users (46). Power relationships are shaped by various forms of oppression that exist within individual attitudes, social systems, and cultural norms (10). Oppression leads to limited opportunities for personal development, restricted ability to participate fully in society, and unequal rights compared to those in dominant groups (10). Immigrant parents, particularly those who lack language proficiency or are unfamiliar with the healthcare system, face heightened vulnerability in clinical and transcultural encounters, making it harder to access care and communicate effectively with professionals.

Adopting an anti-oppressive approach (9, 10) means acknowledging power relations at the personal, systemic, and cultural levels. This requires professionals and researchers to engage in critical reflection, examine power inequalities within clinical settings at the micro level, and to analyze broader impacts at the exo or macro level (e.g., the experience of oppression from the institutional system or the immigration journey), and actively find ways to empower parents and promote their participation.

Once professionals develop a more profound understanding of power dynamics and the constraints encountered by immigrant parents, they will be better equipped to counteract systemic barriers. In practice, committing to an anti-oppressive approach within the CPPP framework encourages professionals working with immigrant parents to critically examine various forms of oppression at different levels, as well as to counter these forms of oppression. To illustrate, professionals may offer information in accessible language (i.e., the family’s preferred language) and advocate for organizational accommodations (e.g., flexible scheduling or provisions for specific interventions, or accompanying parents to community services). Furthermore, professionals can promote shared decision-making by collaboratively establishing intervention objectives with the family, rather than imposing standardized goals. In other words, professionals can facilitate a more effective response to the needs of these families by adopting an anti-oppressive approach.

This broader interpretation of the framework, blended with the anti-oppressive approach and the transcultural perspective, is crucial in a multi-ethnic, multi-linguistic and multi-cultural societies such as Canada. Immigrant families undergo a transformative process (47). This reality, filled with disruptions and changes in family life calls for a perspective sensitive to cultural transitions, political ideologies, and unique journeys of immigrant families. The adapted framework proposes considering the macro and exo-levels representing dominant beliefs, ideologies and policies that directly or indirectly affect the CPPP. Emphasizing an anti-oppressive approach in practice is essential for fostering genuine partnerships and addressing the complexities faced by immigrant families.

## 8 Implications for practice, research and policy

This guiding conceptual framework proposes a paradigm shift from the concept of parental engagement to a more nuanced term that best represents the relationship that occurs in EI services. The CPPP could be tested both in research settings and in intervention contexts. It was conceptualized for the first author’s doctoral project, which will use a case study methodology mixed with an intervention-research approach to examine the CPPP under real-life conditions; where parents and professionals partner to generate ideas for improving services for immigrant families in a provincial healthcare program.

In fact, this proposed conceptual framework carries profound implications for practice and research in the domain of EI. It has been developed to promote reflective practice among professionals and researchers usually working in a multidisciplinary team. The CPPP emphasizes a transition toward more inclusive, reflective, and partnership-oriented models of care and offers a comprehensive view of the influences affecting engagement in a transcultural context.

### 8.1 Implications for practice

The new conceptual framework based on the bioecological model, provides a holistic ecological perspective on immigrant families and professionals, their conditions, and related implications in this dynamic context. By considering the complex interplay of individual, social and systemic factors, practitioners can tailor their interventions more effectively, thereby increasing their relevance and impact. For example, the CPPP facilitates the mapping of available support networks (i.e., extended family members or ethnic community, community-based services). It could also reveal the precarious and unstable status of certain families, such as asylum seekers with medical complications and no access to the health and social services. Moreover, this framework could uncover the challenges encountered by professionals in their personal or professional lives (i.e., health or family concerns, workplace conflicts, policy reform and implementation), as well as those arising from the distal context (i.e., sociocultural norms, political discourse about immigration). These factors significantly influence professional interactions within transcultural contexts, warranting careful attention and consideration. In other words, the CPPP enables professionals to reflect not only on factors related to the family but also to reflect on their own conditions in the relational dynamic. It fosters equitable and reciprocal relationships with immigrant families in a transcultural context. Rather than viewing PE as mere participation, that needs to be monitored, the CPPP encourages professionals to view the relationship as a collaborative dynamic process that values the unique expertise and experiences of each partner (family members and professionals).

In addition, to providing insights into the broader context, the framework offers guidance on the adoption of an anti-oppressive approach that fosters authentic partnerships and reduces power imbalances. Rooted in social work principles, this approach calls for incorporating reflexivity concerning social positioning, professional posture and power dynamics in practice. This is pivotal in advocating for a relationship characterized by horizontal collaboration, thus challenging traditional hierarchical dynamics. Anti-oppressive approach forms the foundation for authentic partnerships and is instrumental in promoting empowerment and ensuring interventions are culturally sensitive and contextually appropriate.

### 8.2 Implications for research

The CPPP was developed as a heuristic tool designed to simplify and facilitate understanding of complex concepts. Its objective is threefold: (1) to help the researcher organize key concepts in preparation for the empirical work, (2) to identify relationships between these concepts, and (3) to stimulate research. As previously stated, this framework serves primarily as a guide for the first author’s exploratory research activities. It is not an established theory, but rather an adaptable formula intended to generate ideas and guide future empirical research.

At this stage, the implications of this innovative framework for research are limited in scope due to an absence of empirical testing that will be addressed in the subsequent stage of the first author’s doctoral project. However, the CPPP provides a broad perspective, enabling researchers to reflect on the different levels of context (ranging from micro to macro and including the transcultural context) that influence families, professionals, and intervention outcomes. Moreover, the anti-oppressive lens invites researchers to direct greater attention to the subtle power dynamics component, which has the potential to influence the development of a genuine partnership. For instance, in the context of participatory research, this framework can assist researchers in ascertaining the presence of a genuine equitable relationship and the nature of the collaborative endeavor. In a case study involving a CPP partnership, it may help identify the multiple factors that played a role in the intervention for one or more partners (e.g., professionals or parents).

### 8.3 Implications for policy

The proposed theoretical framework challenges rigid institutional structures that are often organized in silos with strictly defined mandates within different departments. By promoting collaboration and an anti-oppressive lens, the framework invites us to reconsider traditional operating methods that reproduce forms of oppression toward both the people who use the services and the professionals who work there. This perspective highlights the need to reevaluate mandates, regulations, and restrictions, and to critically reflect on the culture of performance and productivity that shapes intervention contexts. Creating more flexible, interconnected environments that focus on the real needs of partners is essential to promoting more equitable, humane, and effective practices.

Furthermore, the framework emphasizes the importance of reducing the separation between institutional services and those offered by community organizations. Community organizations play a crucial role in supporting families and children, often complementing public services. Greater openness, recognition, and integration of community resources into service pathways would strengthen partnerships, ensure continuity of care, and better respond to populations’ complex needs.

## 9 Limitations of this work

It is imperative to acknowledge the limitations inherent to this work, which influence its transferability and generalizability. These limitations can be categorized into those that pertain to the methodology and those that pertain to researcher bias during the process, which are intertwined.

Due to its preliminary nature and as the proposed framework is the result of a critical literature review that adopted a non-systematic approach during its process, further empirical studies are required to test its resonance in intervention and in research. A non-systematic approach, in which multiple sources are triangulated, has the potential to enhance the validity of the research. However, it is important to acknowledge the potential risks in such an endeavor, including the possibility of researcher bias. Therefore, we recognize the inherent subjectivity that could have resulted in potential bias in the selection of articles and analysis presented here. The inclusion of only 30 articles and a 10-year frame may result in the omission of other pertinent research and models outside of the specified period. Furthermore, the focal point of the review was on PE, we acknowledge the need to expand understanding to include the circumstances of professionals and other potential partners. The article provides a comprehensive overview but cannot address all aspects within its scope. Finally, we recognize the importance of expanding the framework to encompass a wider range of factors affecting the Child–Parent-Professional Partnership.

## 10 Conclusion and recommendations for future research

This article presents a preliminary theoretical framework developed through a critical review of models and research on PE. A key contribution of this work lies in its foundation in social work, drawing on diverse disciplinary perspectives to inform researchers and professional practice. By adopting a holistic approach that bridges theoretical approaches, the framework aims to promote more inclusive, culturally sensitive, and effective EI practices that truly empower families and optimize outcomes for children with NDC. Viewing the complexities of immigrant parents’ realities when viewed through the lens of transcultural context and power dynamics provides a broader understanding of the factors affecting the CPPP. By considering contributions from all actors and the broader social environment, it promotes reflection on the influences, challenges and opportunities within interpersonal relationships. The framework provides guidance for critical reflective practice, pivotal to unveiling and potentially reducing power dynamics in EI. This approach is particularly beneficial when establishing equitable partnerships with immigrant families, as it encourages greater consideration of how the broader social environment impacts partner interactions. The framework is a valuable tool for both researchers and professionals, offering a representation of micro and macro elements that influence engagement.

The robustness and resonance of this framework in its application are currently being analyzed. Since the development of this new conceptual model, the first author has designed and conducted research in an EI rehabilitation program uniting immigrant parents and child development professionals working in a provincial EI program. A series of group meetings, spanning a period of 6 months, culminated in the co-construction of a set of recommendations that drew upon the insights of both parents and professionals. Subsequent publications will present findings on the implementation of the model. In other words, these publications will detail how the insights derived from this framework were utilized to design the group intervention and to analyze the intervention’s outcomes.

The framework also holds potential application for other settings, such as community service, rehabilitation programs and schools. By integrating an anti-oppressive practice approach, this model seeks to equip researchers and professionals with strategies to recognize and address power imbalances within child–parent-professional interactions, fostering authentic and equitable partnerships. Additionally, it enables the exploration of immigrant parents’ preoccupations, values, and realities from a transcultural perspective, raising awareness of their migratory journeys, including traumas, losses and cultural knowledge - and their impact on parenting. Acknowledging these realities is essential for building an authentic CPPP.

## Data Availability

The original contributions presented in the study are included in the article/Supplementary material, further inquiries can be directed to the corresponding author.

## Footnotes

1 “I define a theory as a generalized set of ideas that describes and explains our knowledge of the world around us in an organized way. A social work theory is one that helps us to understand and to do social work. A social work practice theory describes and explains what actions to take when doing social work” (4), p. 3.

2 Early intervention is defined as: “the provision or mobilization of supports and resources to families of young children from informal and formal social network members that directly or indirectly influence and improve parent, family, and child behaviour and functioning in ways having capacity-building consequences” (48), p. 37.

3 The year 2020 was initially selected as the endpoint for the literature review, as it coincided with the comprehensive examination. In order to guarantee the relevance and recency of the included studies, the search was then extended retrospectively over a 10-year period, commencing in 2010.

4 “Reflectivity is the process of recognizing how your own assumptions and actions contribute to a situation. … Reflexivity is described as one’s positioning complemented by act of being reflective” (45), p. 42.
