# Supplementary material for: Proposal for a new conceptual framework to guide early intervention professionals in a transcultural context
Source: Front Public Health. 2025 Dec 12;13:1689596. doi: 10.3389/fpubh.2025.1689596 (PMC12741139; doi:10.3389/fpubh.2025.1689596)
Supplement: Supplementary file 1 [file Data_Sheet_1.pdf]

Supplementary Table 1. Models presented in the selected articles

	Models identified in the selected literature	Reference
1	*Conceptual model of parent involvement*<br><br>- Setting: **family support programs**<br>- Originality:<br>o The model is anchored in ecological and family systems frameworks.<br>o Engagement influenced by factors at different levels: individual/family, provider attributes, program characteristics, and neighborhood characteristics.<br><br>- **When mapped into the bioecological model,** we interpreted the different levels mentioned as follow: individual and family factors (micro level), provider attributes (meso), program and neighborhood (exo level).	McCurdy, K., & Daro, D. (2001). Parent Involvement in Family Support Programs : An Integrated Theory*. *Family Relations*, *50*(2), 113121. https://doi.org/10.1111/j.1741-3729.2001.00113.x
2	*Conceptual framework of the engagement process*<br><br>- Setting: **treatment engagement with caregivers of at-risk children** (mental health and other services)<br>- Originality:<br>o Engagement is an ongoing process.<br>o Engagement has two components: behavioral (e.g., attendance, participation) and attitudinal (e.g., commitment, motivation).<br>o Barriers to engagement at different levels: child, family, agency, neighborhood, community.<br><br>- **When mapped into the bioecological model,** we interpreted the different levels as follow: child, family factors (micro level), agency, neighborhood and community factors (exo level).	Staudt, M. (2007). Treatment Engagement with Caregivers of At-risk Children : Gaps in Research and Conceptualization. *Journal of Child and Family Studies*, *16*(2), 183196. https://doi.org/10.1007/s10826-006-9077-2
3	*Model of factors acting as barriers to parental involvement*	Hornby, G., & Lafaele, R. (2011). Barriers to parental involvement in education : An

	- Setting: **Education**<br>- Originality:<br>o The model identifies factors acting as barriers.<br>o Barriers operate at multiple levels: parent and family factors**,** child factors**,** parent–teacher factors and societal factors (historical, demographic, economic and political)**.**<br>o One of the few that considers the societal factors (macro level).<br><br>- **When mapped into the bioecological model**, we interpreted the different levels as follow: parent/family and child factors (micro), parent–teacher (meso) and societal (macro).	explanatory model. *Educational Review*, *63*(1), 3752. https://doi.org/10.1080/00131911.2010.488049
4	*Integrated model of parental engagement with child welfare services. (developed from ward et al. 2004)*<br><br>- Setting: **Child welfare**<br>- Originality:<br>o Engagement is a dynamic process<br>o The model employs an ecological approach and integrates structural, relational, and motivational components.<br>o Engagement influenced by multiple factors: internal determinants (e.g. cognitive, affective, behavioral, identity, motivation), external determinants (e.g. program, resources etc.), interactional factors (working alliance) and background (social and other facors).<br>o One of the few studies that considered background factors (macro-level) in a flexible way, while also focusing on more proximal factors.<br><br>- **When mapped into the bioecological model**, we interpreted the different levels as follow: Internal determinants (micro level) and interactional (meso level), external (exo level) and background (macro level)	Platt, D. (2012). Understanding parental engagement with child welfare services : An integrated model. *Child & Family Social Work*, *17*(2), 138148. https://doi.org/10.1111/j.1365-2206.2012.00828.x
5	*A multidimensional framework for patient and family engagement in health and health care*	Carman, K. L., Dardess, P., Maurer, M., Sofaer, S., Adams, K., Bechtel, C., &

	- Setting: **Health and health care**<br>- Originality:<br>o Engagement as a continuum of involvement from consultation to partnership and shared leadership.<br>o Conceptualizes engagement across three levels: direct care, organizational design and governance, and policymaking.<br>o Core elements: respect, transparency, and shared decision-making, and cultural change within health systems.<br><br>- **When mapped into the bioecological model**, we interpreted the different levels as follow: direct care (micro level), organizational design and governance and policymaking (exo level).	Sweeney, J. (2013). Patient And Family Engagement : A Framework For Understanding The Elements And Developing Interventions And Policies. *Health Affairs*, *32*(2), 223231. https://doi.org/10.1377/hlthaff.2012.1133
6	*Continuum from involvement to engagement*<br><br>- Setting: **Education**<br>- Originality:<br>o Engagement as a continuum that distinguishes parental involvement (supporting school-led activities) from parental engagement (active collaboration in children's learning).<br>o The model emphasises a shift from school agency to parental agency within this continuum; moving from passive participation to meaningful partnership, where parents assume the roles of co-educators and decision-makers.<br>o The model highlights the importance of shared responsibility and mutual respect in educational processes.<br><br>- **When mapped into the bioecological model**, we interpreted the levels as follow: engagement (micro level) and involvement (meso level)	Goodall, J., & Montgomery, C. (2014). Parental involvement to parental engagement : A continuum. *Educational Review*, *66*(4), 399410. https://doi.org/10.1080/00131911.2013.781576
7	*Client engagement in the clinical change process*<br><br>- Setting : **Mental Heath**	King, G., Currie, M., & Petersen, P. (2014). Child and parent engagement in the mental health intervention process : A motivational

	- Originality:<br>o Engagement as a motivational process involving both children and parents in clinical settings.<br>o Components of engagement: affective, cognitive and behavioral<br>o Engagement influenced by factors at different levels: personal, relational, and contextual.<br><br>- **When mapped into the bioecological model**, we interpreted the different levels as follow: personal factors (micro level), relational factors (meso level) and the contextual factors (exo level). The text does not allude to the broader macro.	framework. *Child and Adolescent Mental Health*, *19*(1), 28. https://doi.org/10.1111/camh.12015
8	*What is the key to engagement?*<br><br>*"a graphical representation of how Self-Determination Theory (SDT) elements (autonomy, relatedness and competence) contribute to the process of 'engaging with' and the state of 'engaged in' therapy (affective, behavioural and cognitive manifestations) in order to achieve an optimal engagement." (p.342)*<br><br>- Setting: **Therapy (occupational)**<br>- Originality:<br>o The model uses Self-Determination Theory (SDT) to explain optimal engagement in therapy.<br>o Three psychological needs drive the process of engagement : autonomy, relatedness, and competence.<br>o Engagement expressed through affective, behavioral, and cognitive dimensions.<br><br>- **When mapped into the bioecological model**, we interpreted that the focus in this model is more at the micro level.	D'Arrigo, R., Ziviani, J., Poulsen, A. A., Copley, J., & King, G. (2017). Child and parent engagement in therapy : What is the key? *Australian Occupational Therapy Journal*, *64*(4), 340-343. https://doi.org/10.1111/1440-1630.12279
9	*CAPE Model of parental engagement*<br><br>- Setting: **Child mental health**	Piotrowska, P. J., Tully, L. A., Lenroot, R., Kimonis, E., Hawes, D., Moul, C., Frick, P. J., Anderson, V., & Dadds, M. R. (2017).

	- Originality:<br>o The CAPE model conceptualizes parental engagement as a four-stage process :<br>▪ Connect: establishing initial contact and trust<br>▪ Attend: ensuring consistent participation in sessions<br>▪ Participate, active involvement in therapeutic activities<br>▪ Enact, applying strategies at home and sustaining change.<br>o This model highlights engagement as progressive and multidimensional, requiring attention to relational, motivational, and practical factors.<br><br>- **When mapped in the bioecological model**, we interpreted the different levels as follow: motivational (micro level), relational and practical (meso level).	Mothers, Fathers, and Parental Systems : A Conceptual Model of Parental Engagement in Programmes for Child Mental Health—Connect, Attend, Participate, Enact (CAPE). *Clinical Child and Family Psychology Review*, *20*(2), 146161. https://doi.org/10.1007/s10567-016-0219-9
10	*Developmental model of dimensions of father involvement and processes of influence on child outcomes*<br><br>- Setting: **Childhood neurodisability**<br>- Originality:<br>o The model conceptualizes father involvement as multidimensional, including engagement, accessibility, and responsibility.<br>o Involvement is a process that involve direct interaction, financial and emotional support, and role modeling.<br>o Factors that influence the involvement are developmental and contextual (such as family structure and child neurodisability).<br><br>- **When mapped in the bioecological model**, we interpreted the different levels as follow: developmental (micro level) and contextual (meso and exo level). It is unclear whether the macro level is being considered in these contextual factors.	Bogossian, A., King, G., Lach, L. M., Currie, M., Nicholas, D., McNeill, T., & Saini, M. (2019). (Unpacking) father involvement in the context of childhood neurodisability research : A scoping review. *Disability and Rehabilitation*, *41*(1), 110124. https://doi.org/10.1080/09638288.2017.1370497
11	*The Phoenix theory of attendance, participation and engagement.*	Phoenix, M., Jack, S. M., Rosenbaum, P. L., & Missiuna, C. (2019a). A grounded theory

	- Setting: **Developmental rehabilitation services**<br>- Originality:<br>o The Phoenix Theory conceptualizes parental involvement in three interconnected processes:<br>▪ **Attendance:** being physically present at services,<br>▪ **Participation:** active involvement during sessions, and<br>▪ **Engagement:** deeper emotional and cognitive investment in the therapeutic process.<br>o Factors that influence the involvement are contextual (family circumstances, service accessibility) and relational (trust, collaboration)<br><br>- **When mapped into the bioecological model**, we interpreted the different levels as follow: the relational factors (meso level), the contextual factors (exo level). It is unclear whether the macro level is being considered in these contextual factors.	of parents' attendance, participation and engagement in children's developmental rehabilitation services : Part 2. The journey to child health and happiness. *Disability and Rehabilitation*, *0*(0), 110. https://doi.org/10.1080/09638288.2018.1555618<br><br>Phoenix, M., Jack, S. M., Rosenbaum, P. L., & Missiuna, C. (2019b). Parents' attendance, participation and engagement in children's developmental rehabilitation services : *Part 1. Contextualizing the journey to child health and happiness*. *Disability and Rehabilitation*, 110. https://doi.org/10.1080/09638288.2018.1555617
12	*Engagement continuum as defined by coalition for research on engagement and well-being*<br><br>- Setting: **Home visiting**<br>- Originality:<br>o Engagement in home visiting as a continuum ranging from initial enrollment to active participation and sustained involvement.<br>o Engagement is multidimensional covering attendance, relationship quality, and implementation of strategies at home.<br>o Authors identifies systemic challenges such as inconsistent definitions and measurement across models and calls for standardized approaches to improve engagement and program effectiveness.	Guastaferro, K., Self-Brown, S., Shanley, J. R., Whitaker, D. J., & Lutzker, J. R. (2020). Engagement in Home Visiting : An Overview of the Problem and How a Coalition of Researchers Worked to Address this Cross-model Concern. *Journal of Child and Family Studies*, *29*(1), 410. https://doi.org/10.1007/s10826-018-1279-x

	- **When mapped into the bioecological model**, we interpreted the different levels as follow: attendance and implementation of strategies (micro level), relationship quality (meso level)	

Supplementary Table 2. Trends in the exploration of PE found in the selected literature

#	Themes	Elements identified in the selected literature	References
**1**	**Parental engagement studied in disciplinary siloes**	• The concept of PE has been examined by various academic disciplines from their respective perspectives.<br>• A broad variability in terminology is used across different disciplines.<br>• Variability in definitions across these different disciplinary perspectives.<br>• Variability in conceptual models.	Carman et al., 2013; D'Arrigo et al., 2018; Goodall & Montgomery, 2014; Hornby & Lafaele, 2011; King et al., 2014; McCurdy & Daro, 2001; Phoenix et al., 2019; Piotrowska et al., 2017; Platt, 2012; Staudt, 2007
**2**	**Narrow focus on factors that affect PE by mapping on the bioecological model**	• PE primarily examined at the micro and meso levels, primary focus on the interaction between the parent and the professional.<br>• Limited attention to exo level of bioecological model.<br>• A limited number of studies presented elements on the macro level of bioecological model.	Burns et al., 2014; Carman et al., 2013; Hornby & Lafaele, 2011; McCurdy & Daro, 2001; Phoenix et al., 2019; Stadnick et al., 2016; Waid & Kelly, 2020
**3**	**Variability in delineating PE**	• PE has been studied, revealing the barriers and benefits in different programmes and contexts.<br>• A series of models have been developed to describe PE and its components using different terminology (i.g. dimensions, conditions).	Burns et al., 2014; Gopalan et al., 2010; Hornby & Lafaele, 2011; King et al., 2014; Phoenix et al., 2019; Piotrowska et al., 2017; Platt, 2012; Rivard et al., 2019

Supplementary Table 3. Characteristics of PE found in the selected literature

	Themes	Characteristics of PE	References
1	**Process-oriented and multifaceted nature**	• PE is a dynamic process<br>• Importance of considering factors beyond simple indicators of attendance,<br>• PE has a multifaceted set of conditions for example personal factors, programme-related factors and societal factors<br>• Two main actors involved: the practitioner and the parent.	Burrell & Borrego, 2012; D'Arrigo et al., 2017; Goodall, 2015; Gopalan et al., 2010; Guastaferro et al., 2020; King et al., 2014; Burns et al., 2014; Phoenix et al., 2019; Platt, 2012; Staudt, 2007.
2	**Contextual and complex interactive dynamics**	• Interactions within the child's environment have a significant impact on intervention outcomes.<br>• Most articles focus on two main actors and the interaction between them: the practitioner and the parent.<br>• Complex interactions occur in the ecosystem of the child, such as the interparental dynamic and other family conditions.	Burns et al., 2014; King et al., 2014; Phoenix et al., 2019; Piotrowska et al., 2017; Platt, 2012; Staudt, 2007.
3	**Continuum of engagement**	• PE as a continuum.<br>• PE change/ develop with time.<br>• Actions and responsibilities within PE change progressively and are renewable and are adaptable to the development of the child and their evolving needs.	Carman, 2013; D'Arrigo et al., 2018; Goodall, 2015; Hoover-Dempsey et al., 2005.
4	**Partnership based approach**	• Engagement as a partnership.<br>• Engagement with a democratic approach highlighting the role of the professional and the child's role.	Rivard et al., 2020; Roose, 2013.

Supplementary Table 4. Limitations and sources of inspiration for developing the CPPP framework

Key players	Reciprocal interdependence	Contextual framework	Representation of experiences	Terminology and Responsibility
**Limitations:**<br>- The scope of discussion is constrained to the parent and the professionals' factors (mostly personal and interactional level).<br>- Elements associated with other primary actors, such as factors associated with the child and extended family (i.e: siblings, grandparents, fathers) are also not considered.	**Limitations:**<br>This limited focus on two main actors (parents & professionals) results in an inadequate representation of the complex dynamic of interactions between various stakeholders.	**Limitations:**<br>When mapped onto the bioecological model, we see that the contextual framework was narrower and focused more on the micro, meso, and sometimes exo levels, thereby missing the macro level.	**Limitations:**<br>- Oversimplification of the complex experiences of families with the<br>- Overlooking the unique experiences of immigrant families.	**Limitations:**<br>The term 'parental engagement' places the burden of collaboration on only one actor in this complex dynamic of interaction: the parent.
**Sources of inspiration for developing the CPPP framework:**<br>- Expand the vision to include more than the two main partners in this dynamic.<br>- Family constellations are diverse. So, be	**Sources of inspiration for developing the CPPP framework:**<br>- Consider the different dyads and triads that could influence the partnership (with an expanded vision that	**Sources of inspiration for developing the CPPP framework:**<br>- Incorporate the sociocultural macro-level, encompassing the transcultural context.	**Sources of inspiration for developing the CPPP framework:**<br>- Expand the scope to incorporate the integration of the diverse experiences of immigrant families and	**Sources of inspiration for developing the CPPP framework:**<br>- Questioning the use of this term.<br>- Selecting a term that more accurately

flexible about who can be included in response to families' unique practices and beliefs.<br>- Consider other professionals who play a role in this dynamic but are sometimes overlooked, especially if they are not part of the same institution (e.g. community organization stakeholders)	includes more than the two main partners).<br>- Consider that these partnerships may be on a second level of interaction or not visible at the micro level. For example, consider the parent-parent alliance that Piotrowska discussed.<br>- Consider also professional interactions (professional – professional relation) that can occur in multidisciplinary teamwork, as well as those affiliated with different institutions or organizations.	- Allocate greater attention to the power dynamics within such environments.	the transcultural and anti-oppressive perspectives. This perspective encompasses the immigration journey and the power dynamics aspects.	represents the type of interaction that must occur in the intervention.
